# Potential ecological risk assessment and predicting zinc accumulation in soils

**DOI:** 10.1007/s10653-017-9924-7

**Published:** 2017-02-22

**Authors:** Agnieszka Baran, Jerzy Wieczorek, Ryszard Mazurek, Krzysztof Urbański, Agnieszka Klimkowicz-Pawlas

**Affiliations:** 1 Department of Agricultural and Environmental Chemistry, University of Agriculture in Krakow, Al. Mickiewicza 21, 31-120 Krakow, Poland; 2 Department of Soil Science and Soil Protection, University of Agriculture in Krakow, Al. Mickiewicza 21, 31-120 Krakow, Poland; 3 AGH University of Science and Technology, Al. Mickiewicza 30, 30-059 Krakow, Poland; 4 Department of Soil Science Erosion and Land Protection, Institute of Soil Science and Plant Cultivation – State Research Institute, ul. Czartoryskich 8, Pulawy, Poland

**Keywords:** Zinc, Geostatistics, PCA, Bioavailable forms of zinc, Soil–zinc binding ability, Risk assessment

## Abstract

The aims of this study were to investigate zinc content in the studied soils; evaluate the efficiency of geostatistics in presenting spatial variability of zinc in the soils; assess bioavailable forms of zinc in the soils and to assess soil–zinc binding ability; and to estimate the potential ecological risk of zinc in soils. The study was conducted in southern Poland, in the Malopolska Province. This area is characterized by a great diversity of geological structures and types of land use and intensity of industrial development. The zinc content was affected by soil factors, and the type of land use (arable lands, grasslands, forests, wastelands). A total of 320 soil samples were characterized in terms of physicochemical properties (texture, pH, organic C content, total and available Zn content). Based on the obtained data, assessment of the ecological risk of zinc was conducted using two methods: potential ecological risk index and hazard quotient. Total Zn content in the soils ranged from 8.27 to 7221 mg kg^−1^ d.m. Based on the surface semivariograms, the highest variability of zinc in the soils was observed from northwest to southeast. The point sources of Zn contamination were located in the northwestern part of the area, near the mining–metallurgical activity involving processing of zinc and lead ores. These findings were confirmed by the arrangement of semivariogram surfaces and bivariate Moran’s correlation coefficients. The content of bioavailable forms of zinc was between 0.05 and 46.19 mg kg^−1^ d.m. (0.01 mol dm^−3^ CaCl_2_), and between 0.03 and 71.54 mg kg^−1^ d.m. (1 mol dm^−3^ NH_4_NO_3_). Forest soils had the highest zinc solubility, followed by arable land, grassland and wasteland. PCA showed that organic C was the key factor to control bioavailability of zinc in the soils. The extreme, very high and medium zinc accumulation was found in 69% of studied soils. There is no ecological risk of zinc to living organisms in the study area, and in 90% of the soils there were no potentially negative effects of zinc to ecological receptors.

## Introduction

Nowadays, there are global concerns regarding soil pollution with heavy metals, due to their bioaccumulation, toxicity and inability to degrade (Khan et al. 2013; Liu et al. 2013a; Baran et al. 2014; Czech et al. 2014a; Bortey-Sam et al. 2015; Baran and Wieczorek 2015; Caetano et al. 2016). Heavy metals have been exerting increasing pressure on the soil ecosystem over the past decades because of the intensifying industrial, urban and agricultural activities. Urban soils, rich in heavy metals, directly endanger humans due to their close proximity to human activities, while polluted agricultural soils pose an indirect risk due to possible transfer of heavy metals through the food chain (Sterckeman et al. 2000; Fazeli et al. 2009; Sun et al. 2010; Hani and Pazira 2011; Liu et al. 2013b; Bortey-Sam et al. 2015). Accumulation of heavy metals in soil is of interest because of the adverse effects they may have on food quality, soil health and the environment (Khan et al. 2013; Olaniran et al. 2013; Rutkowska et al. 2015). The risk to the environment and human health associated with heavy metals is a function of their mobility and bioavailability (Kabata-Pendias 2004). Zinc belongs to the most mobile and bioavailable heavy metals in soil; therefore, its high concentrations in the soil solution may have a phytotoxic effect and reduce the crop yield and quality (Reichman 2002; Broadley et al. 2007; Sagardoy et al. 2009; Baran 2012; Liu et al. 2013b; Baran 2013). Moreover, Zn inhibits the growth and alters morphology and metabolism of soil microorganisms (Hani and Pazira 2011; Olaniran et al. 2013). In the soil solution, zinc occurs mainly in the Zn^2+^ form and in organometallic complexes. Several methods of determining bioavailable forms of zinc in soils have been described (Ure et al. 1993; Rauret 1998; Degryse et al. 2003; Pueyo et al. 2004; Meers et al. 2007; Anju and Banerjee 2011; Baran 2012; Fedotov et al. 2012; Baran et al. 2014; Czech et al. 2014b; Kim et al. 2015; Rutkowska et al. 2015). A number of studies have indicated that bioassays are a good tool for the assessment of the ecological risk of zinc (Baran 2013; Baran and Jasiewicz 2013; Baran et al. 2014; Wieczorek and Baran 2013; Lago-Vila et al. 2016; Romero-Freire et al. 2016). Other authors suggest that mapping of the spatial distribution of zinc-polluted soils is necessary for human and ecological risk assessment (Hani and Pazira 2011; Kizilkaya et al. 2011; Liu et al. 2013b; Krami et al. 2013; Delavar and Safari 2016). Guo et al. (2012) reported that information about the pattern of heavy metal distribution in soils with different land use assists in developing strategies to protect the environment and human health against long-term accumulation of metals. Moreover, different physicochemical soil factors such as pH, organic matter content, granulometric composition, iron and manganese hydroxides, oxidation-reducing potential, sorptive capacity and moisture play an important role in the processes of zinc binding and bioavailability in soil (Kabata-Pendias 2004; Kim et al. 2015).

Nowadays, key studies on heavy metals in soils cover potential ecological risk, geochemical cycling, assessment of health risk caused by metals and toxicity assessment. Different environmental factors and methods (chemical, geostatistical and biological) must be taken into account for the assessment of ecological risk caused by exposure to heavy metals in the soil environment. Moreover, all these factors should be integrated. The novel aspect of our study is the integration of different methods in order to assess the environmental risk associated with the presence of zinc in soils.

The aims of this study are: (1) to investigate the content and distribution of zinc in the soils of the studied area; (2) to evaluate the efficiency of geostatistics in presenting spatial variability of zinc in the soils; (3) to assess bioavailable forms of zinc in the soils and soil–zinc binding ability; (4) to analyze a possible relationship between zinc and soil properties; and (5) to estimate the potential ecological risk of zinc in soils.

## Materials and methods

### Study area

**Fig. 1:**
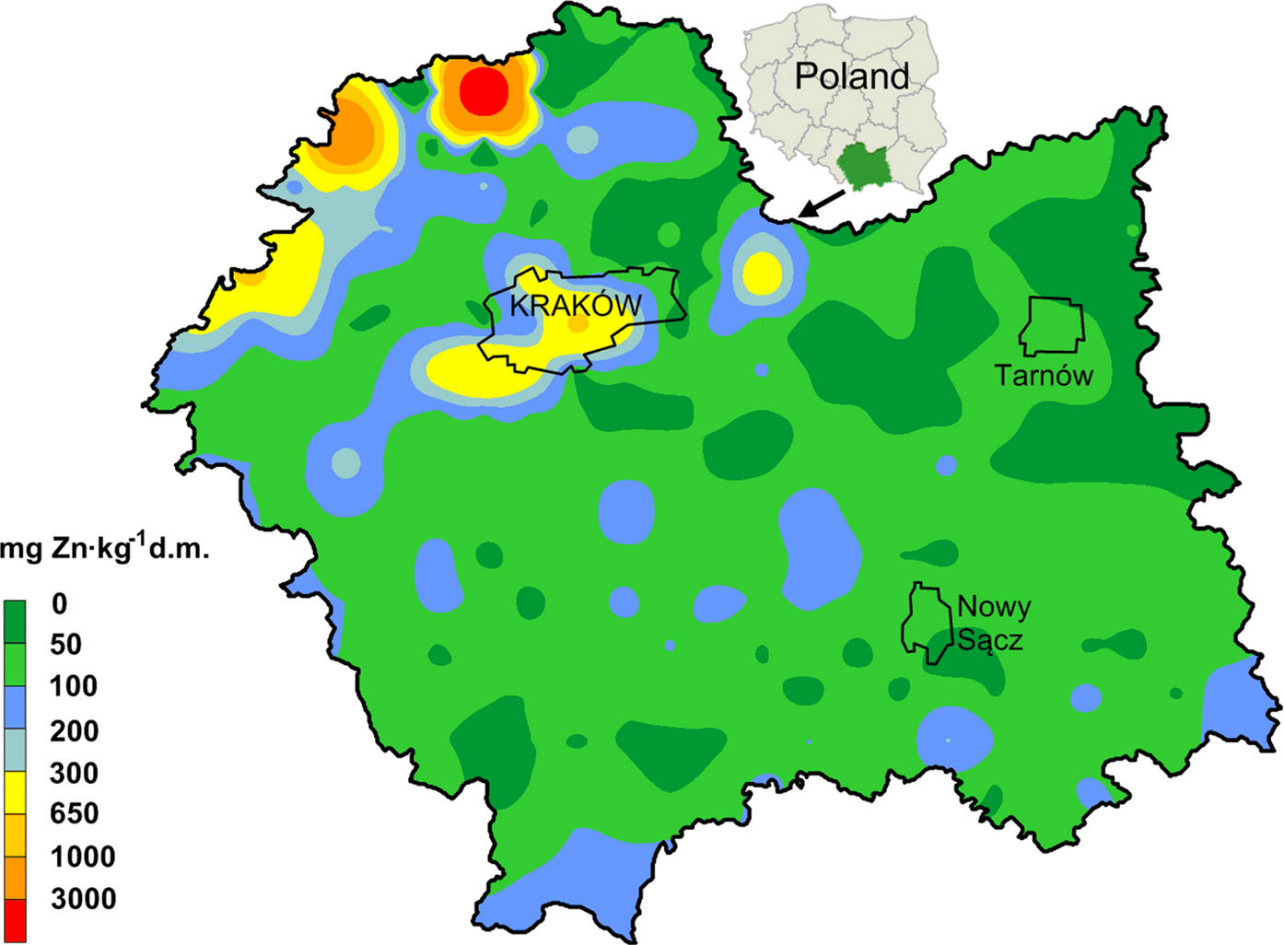
Spatial distribution of total zinc concentrations in the soils (*n* = 320)

The study was conducted in southern Poland, in the Malopolska Province (Fig. 1). The area under investigation is approximately 15.183 km^2^, which is 4.9% of the total area of Poland. It is characterized by a great diversity of geological structures and types of land use and intensity of industrial development (Baran et al. 2014; Baran and Wieczorek 2015). The high environmental variability in the Malopolska Province is caused by differences in altitude in this area—from flat lowlands of the Sandomierz Basin (135–288 m above sea level) to high peaks of the Tatra Mountains (300–2499 m above sea level). A big part of the province lies higher than 500 m a.s.l. There are seven climatic and plant zones, and precipitation is highly diversified. Parent rock and other natural conditions (landform, climate–predominance of precipitation over evaporation, the greatest declivities in the country, acidification and erosion) have an effect on soil fertility variation in the Malopolska Province. In terms of economy, western and central parts (with Krakow agglomeration) of the area are industrialized. Northern and eastern parts have a typically agricultural character, with the exception of two big cities—Tarnow and Nowy Sącz (Fig. 1). Arable lands and forests, which cover up to 92% of the area, are predominating in the land use structure of the province. Urbanized, industrialized areas and areas covered by transport constitute approximately 6% of the Malopolska Province. Soils of moderate and low agricultural capability predominate on 67% of the Malopolska area. Highly fertile agricultural soils (silty and loamy soils) cover approximately 33% of farmlands. The most favorable soil conditions for agricultural purposes can be found in the northern and northeastern part of the province. Soils of the northwestern Malopolska are the most exposed to chemical degradation associated with heavy metal and PAH pollution. The main sources of soil pollution with heavy metals include big industrial plants, transportation, the power industry and burning coal in individual home furnaces. Other factors which influence heavy metal content in soils of the discussed area are the Upper Silesian Industrial Basin (neighboring from the west) and transfer of pollutants associated with this region. The Upper Silesian area is one of the key urban centers in the country. Moreover, it is a region with a great concentration of industry, mainly hard coal mining, electric power industry, transportation, coking and briquetting plants, companies producing machinery, metals, chemicals and building materials (Baran et al. 2016). Additionally, an important source of heavy metals in the northwestern part of the area is the mining–metallurgical activity involving processing of zinc and lead ores (Cabała et al. 2008). Compared to other regions of Poland, soils located in the northwestern part of Malopolska are distinguished by increased concentrations of zinc, cadmium and lead (Cabała and Teper 2007; Baran et al. 2014; Czech et al. 2014a, b; Baran and Wieczorek 2015).

### Sample collection

The sampling points were systematically distributed in the study area, based on a r 7.5 km × 7.5 km regular grid, with the use of a GPS device (Garmin 62 s, accuracy ± 2 m). This method is the most common sampling approach for geostatistical purposes (Delavar and Safari 2016). In total, 320 grid cells (points) were sampled (Fig. 4). At those points, 5–7 soil subsamples (to obtain a representative sample) were collected from the depth of 0–10 cm using an Eijkelkamp sampler. In the forest soil, the organic horizon (forest litter) was removed before sampling. Among the collected soil samples, arable lands constituted 21% (*n* = 66), grasslands 39% (*n* = 126), forests 27% (*n* = 82) and wastelands 13% (*n* = 46). The soil samples were air-dried and sieved through a 2-mm sieve in order to remove large debris, stone, gravel, plant materials and other waste materials.

### Chemical analysis

The soil samples were analyzed in terms of total and bioavailable forms of zinc, soil texture, pH_KCl_ and organic C content. In order to determine the total content of zinc, the soil samples were digested with a 9 cm^3^ mixture (1:3 v/v) of concentrated acids (HCl and HNO_3_), using the wet method in a closed system in a microwave oven (Baran and Wieczorek 2015). The digestion was carried out in accordance with the program, with the following parameters: power: 1400 W; temperature: 240 °C; time to reach the maximum power: 5 min; time on maximum power: 15 min; ventilation time: 5 min; and cooling time: 40 min. Bioavailable forms of zinc were extracted with 0.01 mol CaCl_2_ dm^−3^ and 1 mol NH_4_NO_3_ dm^−3^ (Rauret 1998; Pueyo et al. 2004; Baran et al. 2014). Extraction of available forms of zinc from soils was conducted by a static method consisting in single shaking of the soil samples with a solution at 1:10 (0.01 mol CaCl_2_ dm^−3^) and 1:2.5 (1 mol NH_4_NO_3_ dm^−3^) soil-to-solution ratio, for the extraction time of 2.5 h. The extracts were separated from the solid residue by centrifugation (3000 rpm for 10 min). Zinc content was determined using a Perkin-Elmer Optima 7300 DV—an inductively coupled plasma atomic emission spectrophotometer (ICP-AES). Particle size distribution was determined by the aerometric method. Soil reaction was determined potentiometrically in a suspension of 1 mol dm^−3^ KCl, and the organic C content was determined by the Tiurin method. The soil samples were analyzed in two replications. If the analysis results of those replications differed from one another by more than ±5%, another two analyses of that sample were conducted. The quality of the determinations was verified based on the results of zinc determinations obtained for the internal standard and on the certified reference material CRM023-050. Analytical results of the quality control samples showed good agreement with the certified value; recovery for Zn was 95%.

### Data processing

#### Soil–zinc binding ability

Soil–zinc binding ability was evaluated using the technique suggested by several authors (Blume and Brummer 1991; Towers and Paterson 1997; Wieczorek and Baran 2013). Three soil properties were used: pH, organic C content and clay content. These attributes enable soil classification on a scale from 0 to 5: class 0 means lack of soil–zinc binding ability, whereas class 5 indicates soils with an extreme capacity for zinc accumulation. The method of soil classification into particular classes was presented in the study by Wieczorek and Baran (2013).

#### Potential ecological risk of zinc in soils

Based on the obtained data, a quantitative analysis of the risk of zinc in soil was conducted using two methods: potential ecological risk index and hazard quotient (Håkanson 1980; Hill et al. 2000; Solomon and Sibley 2002; Swartjes et al. 2008; Klimkowicz-Pawlas et al. 2012; Jiang et al. 2014; Bortey-Sam et al. 2015). The potential ecological risk index was calculated to assess the harmful effect of zinc in the studied soils. The potential ecological risk index for zinc was calculated based on the following formula (Håkanson 1980; Fang et al. 2012; Jiang et al. 2014; Bortey-Sam et al. 2015):

$$\mathop E\nolimits_{r}^{i} = \mathop T\nolimits_{r}^{i} \times \mathop C\nolimits_{f}^{i} = \mathop T\nolimits_{r}^{i} \times \frac{{\mathop C\nolimits^{i} }}{{\mathop C\nolimits_{n}^{i} }}$$

where *E*_*r*_^*i*^ is the potential ecological risk of zinc; *T*_*r*_^*i*^ is the toxic-response factor of zinc, *T*_*r*_^*i*^ = 1 (Håkanson 1980; Jiang et al. 2014); *C*_*f*_^*i*^ is the index of zinc pollution, *C* ^*i*^ represents measured values of zinc in the soils; and *C*_*n*_^*i*^ is the background value of zinc in the study area, *C*_*n*_^*i*^ = 48 mg Zn mg kg^−1^ d.m. (Kabata-Pendias and Pendias 2001).

Another method commonly used in the risk assessment, especially at the screening stage of the procedure, is a hazard quotient (HQ). This index is determined by the following equation (Swartjes et al. 2008):

$${\text{HQ}} = \frac{{_{{\mathop C\nolimits_{\text{e}} }} }}{{\mathop c\nolimits_{\text{b}} }}$$

where *C*_e_ is the content of zinc in the soil (exposure content), *C*_b_ is the benchmark value of zinc = 300 mg kg^−1^ d.m. (Journal of Law 2002).

#### Statistical analysis

Statistical analysis involved determination of mean, median, standard deviation, minimum, maximum, skewness, coefficient of variation (CV %) and ANOVA. In order to meet the principles of the analysis of variance (additivity, homogeneity of variance and normality of distribution), the data were subjected to logarithmic transformation prior to the analysis. The differences between means were detected by Tukey’s test at significance level of 0.05. In order to identify the important parameters that affect the zinc content in soil, Pearson’s correlation matrix and principal component analysis (PCA) were used. All statistical analyses were run with Statistica 11 software.

#### Geostatistical analysis

Geostatistical analysis provides a set of statistical tools incorporating the spatial and temporal coordinates of observations in data processing, and this is the reason for its increased use in environmental applications (Saito and Goovaerts 2000; Kizilkaya et al. 2011; Hani and Pazira 2011; Delavar and Safari 2016). The global Moran’s autocorrelation coefficient was calculated for zinc content in soils of the Malopolska Province. Queen contiguity weight matrices were used for calculations. Values of the univariate local indicator of spatial autocorrelation (LISA) were evaluated (Anselin 1995; Anselin et al. 2006). Bivariate Moran’s correlation coefficients were calculated as the relationship between point coordinates and spatial weights of zinc content. Significance of Moran’s autocorrelation coefficients and correlation coefficients was evaluated based on random permutation and comparison at pseudo-p value 0.05 (Anselin 1995). Empirical semivariograms, the main tool for the estimation of spatial variability, were created for zinc content in soils of the Malopolska Province. This index was divided into five types of autocorrelation and expressed as their percentage share in the total number of attempts: N—without autocorrelation, H–H—clusters with high values, L–L—clusters with low values, L–H—low values surrounded by high values (“cold spots”) and H–L—high values surrounded by low values (“hot spots”). Variability maps (surface semivariograms) were elaborated based on omnidirectional semivariograms. Surface semivariograms are helpful in determining directions of the highest and lowest spatial variability of anisotropic soil properties (Hani and Pazira 2011; Mucha and Wasilewska-Blaszczyk 2015). The variability map was created in Surfer 8.0 software. The global and local Moran’s autocorrelation coefficients and bivariate correlation coefficients were calculated using GeoDa 1.4.6 software.

## Results and discussion

### Physicochemical properties of the soils

Some physicochemical properties of soil, such as pH, organic matter content and particle size distribution, are important factors of zinc accumulation. Table 1 shows descriptive statistics of basic soil properties. The pH values of the soils ranged from 2.47 to 7.59, and the mean was 4.97. In the studied area, soils with very acid reaction were dominant (41%), followed by soils with acid (29%), neutral (15%), slightly acid (13%) and alkaline (2%) reactions. Forest soils had the lowest pH value; 89% of soils from those areas had very acid and acid reactions. The mean content of organic C ranged from 0.98 to 320 g kg^−1^ d.m. (Table 1). The highest content of organic C was found in forest soils (75.53 g kg^−1^ d.m.), followed by soils from grasslands (54.67 g kg^−1^ d.m.), wasteland (47.14 g kg^−1^ d.m.) and arable land (28.18 g kg^−1^ d.m.). The content of sand, silt and clay was 0–95, 0–65 and 0–71%, respectively (Table 1). The silt and clay fractions were dominant in samples from grasslands, arable land and wasteland. The highest amounts of sand were found in forest soils. The soils showed high variability in the content of sand (CV = 75%) and organic matter (CV = 77%), medium in pH (CV = 25%) and in clay and silt content (CV = 36%, CV = 40%) (Table 1).

**Table 1:** Basic physicochemical properties of soils (topsoil 0–10 cm) *n* = 320

Parameter		Minimum	Mean	SD	Median	Maximum	Skewness	^a^CV%
Sand	%	0	28	21	21	95	1.22	75
Silt		0	33	14	34	65	−0.10	40
Clay		0	38	13	39	71	−0.14	36
pH		2.47	4.97	1	4.81	7.59	0.36	25
C-organic	g kg^−1^ d m	0.98	53.47	41.1	44.88	320	3.12	77
Total Zn	mg kg^−1^ d m	8.27	122.1	428.8	68.9	7221	14.8	351
Arable land		24.36	97.73a^b^	122.7	63.88	820.3	4.10	126
Grassland		8.27	173.2a	672.7	73.32	7221	9.62	388
Forest soils		18.13	84.27a	68.68	64.28	442.9	4.25	82
Wasteland		20.01	84.71a	60.87	73.64	419.1	2.81	72

^a^CV %—variation coefficient

^b^Means followed by the same letters did not differ significantly at α ≤ 0.05 according to the t-Tukey test

### Content and spatial distribution of zinc in the soils

**Fig. 2:**
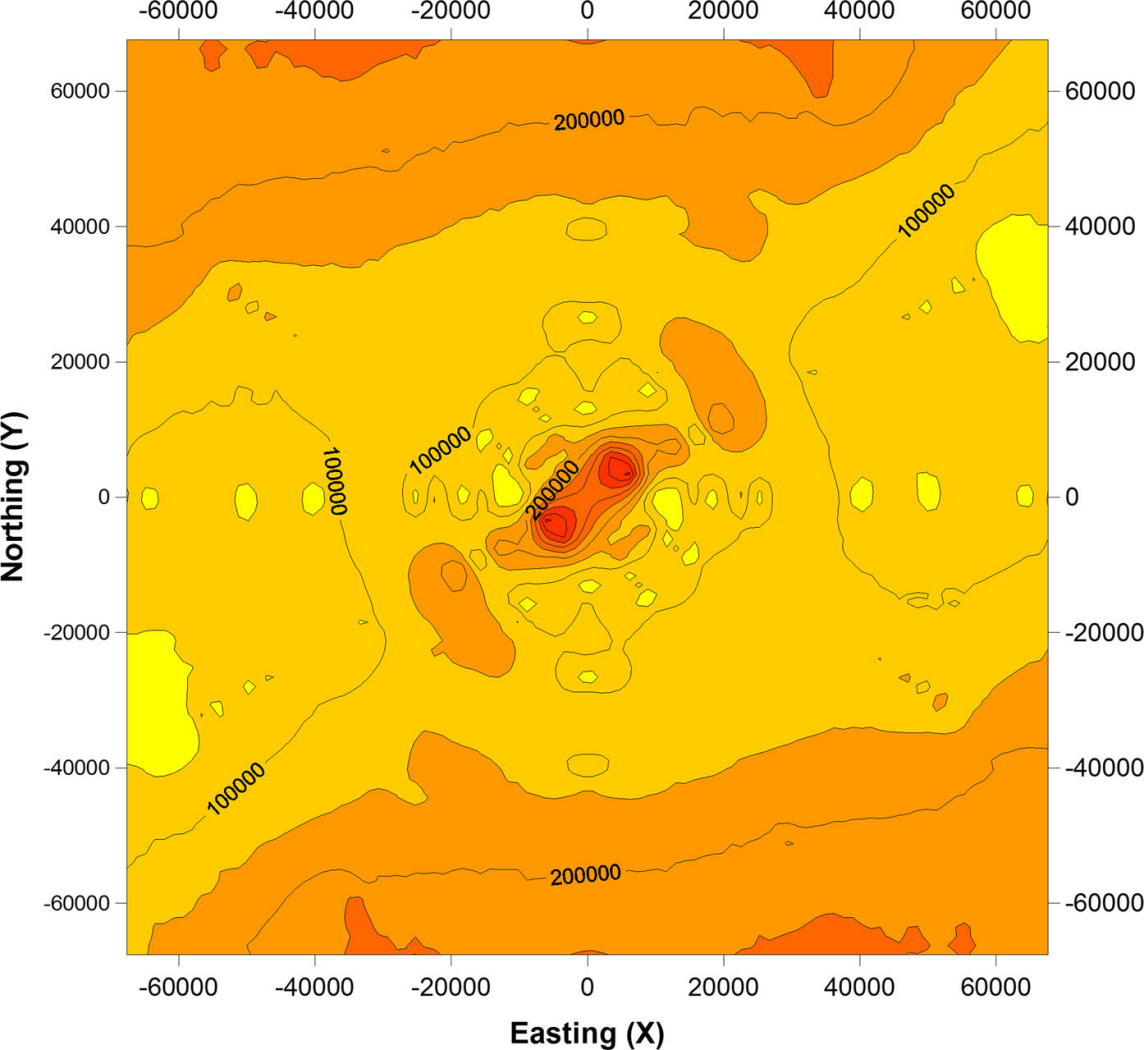
Spatial variability (surface semivariograms) of total Zn in soils of the Malopolska Province

In the soils, considerable changes in total zinc content were observed (Table 1; Fig. 1). Total Zn content varied from 8.27 mg to 7221 mg kg^−1^ d.m. Mean Zn content in the soils was similar to the global mean values for total Zn content (80–120 mg kg^−1^ d.m.) in uncontaminated soils (Kabata-Pendias and Pendias 2001; Alloway 2009). Depending on the type of land use, the mean total zinc content in the soils formed the following order: grassland > arable land > wasteland > forest soils (Table 1). Maximum zinc content in agricultural soils in some European countries may amount up to 300 mg Zn kg^−1^ d.m (Kabata-Pendias and Pendias 2001). In our study, we observed a much higher maximum content of zinc in the soil (Table 1). The total Zn content exhibited a high degree of variability, indicated by high values of coefficients of variation for Zn–351%. Figure 1 shows that the high value of CV reflected the non-homogeneous distribution of zinc content in the study area. Studies of Baran and Wieczorek (2015), Delavar and Safari (2016) showed that CV values of heavy metals originated from natural sources are relatively low, while CV values of heavy metals affected by anthropogenic sources are quite high. Figure 1 shows that higher zinc levels were concentrated in the northwestern part of the study area—the industrial zones, whereas the lowest zinc content in soil was found in the northeastern part of the Malopolska Province. These observations demonstrate that mining–metallurgical activity, which has been conducted for several hundred years, is an important source of zinc distribution in the study area. Moreover, our previous studies showed that heavy metal distribution in soils of the Malopolska Province is strongly affected by various human activities (Baran et al. 2014; Czech et al. 2014a, b; Baran and Wieczorek 2015). The studies showed that zinc content in soils had positive and high values of the skewness coefficient (Table 1). The distribution of zinc content was skewed by a small number of large values. Similar results have been presented by other authors (Kabała et al. 2009; Delavar and Safari 2016). It was stated that global Moran’s index calculated for Zn content was positive and nonsignificant. This proves that, due to a high variability of studied element, spatial clusters with a similar Zn content are absent (Table 2). At the same time, there was a significant dependence between localization of the sampling points and Zn accumulation based on the values of bivariate local Moran’s correlation coefficient. Zinc content was significantly lower with growing distance in the east direction, and higher in the northern direction. Levels of bivariate Moran’s correlation coefficients show that longitude (0.1814) had a greater effect on zinc accumulation in soils than latitude (−0.1507). These findings were confirmed by the share of local autocorrelations. The positive correlation of zinc content with latitude is associated with the highest share (overall more than 10%) of positively correlated points, H–H and L–L, with significant dependences. Moran’s correlation coefficient between Zn content and latitudes of the sampling points shows predominance of “cold spots” (L–H) and “hot spots” (H–L) within LISA (Table 2). The map of spatial variability of zinc content is presented in the form of surface semivariograms (Fig. 2). Based on the surface semivariograms, it can be clearly stated that the highest variability of the studied metal in the Malopolska Province can be observed from northwest to southeast.

**Table 2:** Global and local autocorrelation Moran’s statistics

Dependance		Global Moran’s autocorrelation index (*I*_m_) and bivariate correlation index	Share of local indicator of spatial autocorrelation (LISA) in global autocorrelation (%)				
			N	H–H	L–L	L–H	H–L
Zn							
Lag Zn–Zn		0.0248	77.50	4.06	14.38	4.06	0.00
Lag Zn–Easting		−0.1507*	76.56	0.00	2.50	8.13	12.81
Lag Zn–Northing		0.1814*	78.44	6.56	3.75	0.00	11.25

* Significant at pseudo-value 0.05

The correlation analysis performed on the data enabled the evaluation of the effect of organic C, pH and granulometric composition to control zinc content in soils (Table 3). Total Zn content was significantly positively correlated with pH and organic C (Table 3). Sterckeman et al. (2000) also found significant correlations between organic C content in soil and total zinc. Moreover, Sterckeman et al. (2000) suggested that concentrations of humic acid complexes in the soil solution are linked to organic C content, which thereby facilitates the mobility and bioavailability of zinc. However, no significant correlations were found between the granulometric composition and total zinc content. The lack of correlation between total Zn content and clay may indicate that anthropogenic activities contribute as a source of metals in soils (Shaheen and Rinklebe 2014).

**Table 3:** Relationships between soil properties and content of zinc in soils

Parameters	pH	C-organic	Sand	Silt	Clay	Zn total	Zn (CaCl_2_)	Zn (NH_4_NO_3_)
C-organic	−0.27***							
Sand	−0.04	0.06						
Silt	0.03	−0.12*	−0.78***					
Clay	0.04	0.03	−0.78***	0.23**				
Zn total	0.17**	0.16**	0.07	−0.10	−0.01			
Zn (CaCl_2_)	−0.17**	0.24**	0.18***	−0.14*	−0.15**	−0.01		
Zn (NH_4_NO_3_)	−0.33***	0.33**	0.23***	−0.17*	−0.19***	0.16**	0.54***	

Soil capability for zinc binding, significant at * *p* ≤ 0.05; ** *p* ≤ 0.01; *** *p* ≤ 0.001

### Assessment of bioavailable forms of zinc in the soils

**Fig. 3:**
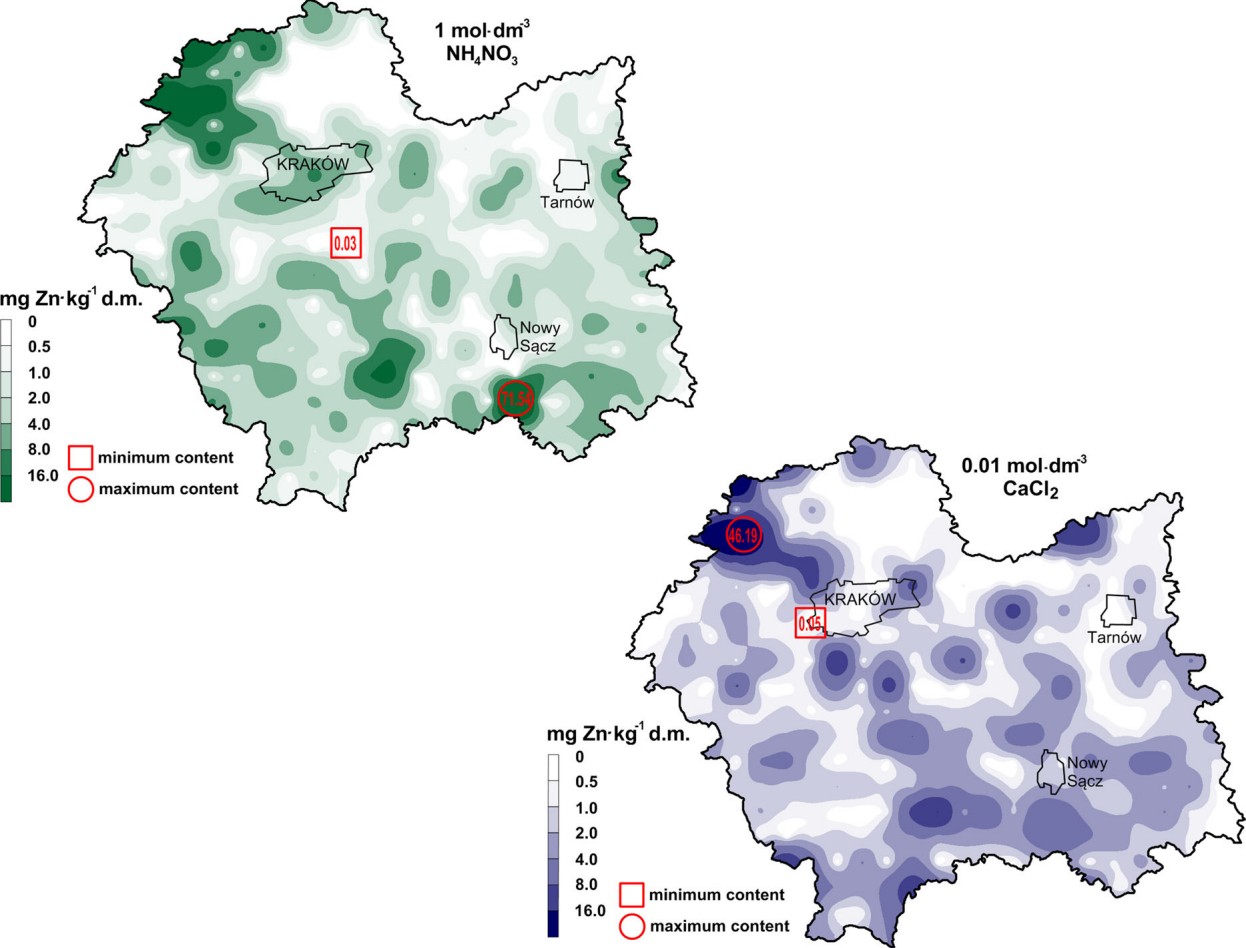
Spatial distribution of the concentration of Zn bioavailable forms in the soils

Total zinc content might serve as a useful indicator of soil contamination. However, it cannot provide sufficient information to assess the environmental impact of contaminated soils because zinc in soils is present in different chemical forms which determine its mobility, bioavailability and potential toxicity (Anju and Banerjee 2011; Shaheen and Rinklebe 2014; Baran et al. 2014; Kim et al. 2015; Rutkowska et al. 2015). The content of bioavailable forms of zinc ranged between 0.05 and 46.19 mg d.m. (CaCl_2_), and between 0.03 and 71.54 mg kg^−1^ d.m. (NH_4_NO_3_) (Fig. 3). Zinc solubility in the soils with respect to its total content ranged from 0.02 to 33.21% (CaCl_2_), and from 0.02 to 75.63% (NH_4_NO_3_) (Table 4). Among the different types of land use, forest soils had significantly the highest mean solubility, followed by arable land > grassland > wasteland in the case of CaCl_2_, whereas grassland > arable land > wasteland in the case of NH_4_NO_3_ (Table 4). These extractants are classified as a solution with low extraction power and an unbuffered neutral salt solution. Numerous authors have found that the use of a neutral salt solution such as CaCl_2_ or NH_4_NO_3_ is adequate to assess the bioavailable forms of zinc, cadmium and nickel (Pueyo et al. 2004; Meers et al. 2007). This is due to the fact that the content of these metals in soils, extracted by the above compounds, is generally well correlated with the response of living organisms (Meers et al. 2007; Baran et al. 2014; Kim et al. 2015). Soils from forest areas were found to have higher amounts of zinc forms soluble in NH_4_NO_3_ than in CaCl_2_ (Table 4). However, soil samples from arable land, grassland and wasteland had an inverse relationship. The study showed that the forest soils generally are very acidic and acidic. Moreover, sand predominated in the granulometric composition of these soils. Most of the soils from forest areas were weakly buffered with a low sorption capacity. According to Kim et al. (2015), NH_4_NO_3_ reduces pH in weakly buffered soils. The decrease in pH in forest soils by this solution may have caused an increase in zinc solubility in these soils. Soil acid reaction is the major factor with the highest impact on zinc mobility and solubility in soils (Rutkowska et al. 2015). This is one of the reasons why some authors have preferred a CaCl_2_ solution over NH_4_NO_3_. Furthermore, the ionic strength of calcium chloride is similar to the one of pore water; Ca^2+^ is better able to displace metals (Zn, Cd) from exchange sites than NH_4_^+^, and low salt concentration reduces analytical interferences (Pueyo et al. 2004; Ettler et al. 2007; Meers et al. 2007; Kim et al. 2015). However, spatial distribution of bioavailable forms of zinc in the soils was similar (Fig. 3). In general, the highest metal concentrations in soils were found in the northwestern and southern part of the Malopolska Province (Fig. 3). Moreover, a high concentration of bioavailable zinc in soils was found also in the northeastern part of the study area (Fig. 3). This study proved that solubility, and consequently bioavailability of zinc, increases at low soil pH. We found a significant negative correlation between soil pH and the content of soluble forms of zinc (Table 3). In their study, Romero-Freire et al. (2016) also showed that pH was significantly negatively correlated with available forms of Zn in soils. The effect of pH on metal solubility and mobility is well known—acidity favors dissolution of Zn, Cd and Ni (Sterckeman et al. 2000; Kim et al. 2015; Romero-Freire et al. 2016). However, despite the significant effect of soil pH, total zinc content also has an important effect on zinc bioavailability. Approximately 30% of the studied area (northwestern part of the Malopolska Province) is exposed to industrial activities connected with processing of zinc–lead ores. Zinc–lead ores can be found in ore-bearing dolomites, which are a source of calcium and magnesium, which in turn have an alkaline effect on the environment (Baran and Wieczorek 2015). Zinc solubility in soils in this area with respect to its total content was, on average, 2.57% (CaCl_2_) and 4.65% (NH_4_NO_3_). Romero-Freire et al. (2016) found that CaCO_3_ content in the soils affected by mining, which is directly associated with soil pH, has an important role in decreasing zinc solubility by precipitation, adsorption and co-precipitation processes. Our previous study showed significantly positive correlations between the total and available zinc content and pH in the soils contaminated by mining (Baran et al. 2014). Numerous authors have found that another physicochemical soil properties play an important role in zinc bioavailability in soils, thus influencing its distribution in soils (Sterckeman et al. 2000; Kabata-Pendias 2004; Baran 2012, 2013; Liu et al. 2013a; Wieczorek and Baran 2013; Olaniran et al. 2013; Bortey-Sam et al. 2015; Rutkowska et al. 2015). This role was illustrated by significant correlations between concentrations of bioavailable forms of zinc in the soils and organic C (positively), sand (positively) and silt and clay content (negatively) (Table 3). Additionally, there was a significantly positive correlation between bioavailable forms of zinc extracted with 1 mol dm^−3^ NH_4_NO_3_ and total zinc content (Table 3). A significantly positive correlation was found between both bioavailable forms of zinc and total zinc only in soil sampled from forest areas (*r* = 0.43 for CaCl_2_ and *r* = 0.38 NH_4_NO_3_, *p* ≤ 0.01).

**Table 4:** Percentage of zinc soluble forms in total content of this metal in soils

Samples	0.01 mol CaCl_2_ dm^−3^			1 mol NH_4_NO_3_ dm^−3^		
	Minimum	Mean	Maximum	Minimum	Mean	Maximum
Arable land	0.03	3.34^a1^	22.55	0.02	1.90^a^	11.31
Grassland	0.02	3.07^a^	31.49	0.05	3.03^a^	75.63
Forest soils	0.11	5.16^b^	33.21	0.08	9.24^b^	46.32
Wasteland	0.09	2.85^a^	16.51	0.04	1.53^a^	12.57
*All samples*	*0.02*	*3.60*	*33.21*	*0.02*	*4.20*	*75.63*

^1^Means followed by the same letters did not differ significantly at α ≤ 0.05 according to the t-Tukey test

Bioavailability in the literature is described in three steps: availability of metals in soil (environmental availability); uptake of metals by organisms (environmental bioavailability); and toxic effect of metals in organisms (toxicological bioavailability) (Harmsen 2007; Kim et al. 2015). Toxic level of zinc in the soil solution is 0.5 mg kg^−1^ (Ewers 1991). Baran (2013) found that zinc content (extracted with 1 mol dm^−3^ HCl) at which a 50% inhibition of *Zea mays* root growth occurred was 1194 mg Zn on sandy soil, and 1320.6 mg Zn kg^−1^d.m. on silty clay soil. In sandy soil, the 50% reduction in *Sinapis alba* root length was found at a zinc content of 183.8 mg Zn, for *Sorghum saccharatum* 246.7 mg Zn, and for *Lepidium sativum* 253 mg Zn kg^−1^d.m. (Baran and Jasiewicz 2013). In unpolluted soil, the Zn EC50 value, calculated in the test of *Lactuca sativa* root elongation and in the soil respiration test, was 4155 mg Zn (2956–5841 mg) and 1842 mg Zn (1234–2749 mg) kg^−1^ dry soil, respectively (Romero-Freire et al. 2016). In our study, a non-specific method using two extraction solutions (NH_4_NO_3_ and CaCl_2_) was applied for the extraction of available forms of zinc from the soil. It is also possible to estimate the content of available forms of zinc in 1 mol dm^−3^ HCl (Baran 2013; Baran et al. 2014). Application of the test with 1 mol dm^−3^ HCl is a routine procedure in Poland, commonly used by chemical–agricultural stations and by the Institute of Soil Science and Plant Cultivation for the assessment of the content of available forms of trace elements in soils. However, hydrochloric acid leaches metals bound to exchangeable, carbonate, Fe/Mn oxides and organic matter fractions. Moreover, as reported by Baran et al. (2014), in the case of heavily polluted soils, metals extraction with such strong substance as 1 mol dm^−3^ HCl does not pose a real risk to living organisms. Currently, there is no uniform data on the method of determining the content of bioavailable forms of zinc in soils. More studies are needed to evaluate the bioavailability of metal pollutants in soils in order to broaden the knowledge on environmental risk assessment (Romero-Freire et al. 2016). Baran (2013) found that toxicity criteria, which indicate whether a given organism is sensitive to soil contamination with metals, should be based on interdependencies between soil and a plant in which biological and chemical effects of metal harmfulness are observed—it is an important key to investigate the bioavailability of metals in soils. Other authors have suggested that free metal activity in the soil solution is the main factor in understanding the availability of metals in the soil environment (Sterckeman et al. 2000; Kim et al. 2015; Rutkowska et al. 2015).

### Soil–zinc binding ability

**Fig. 4:**
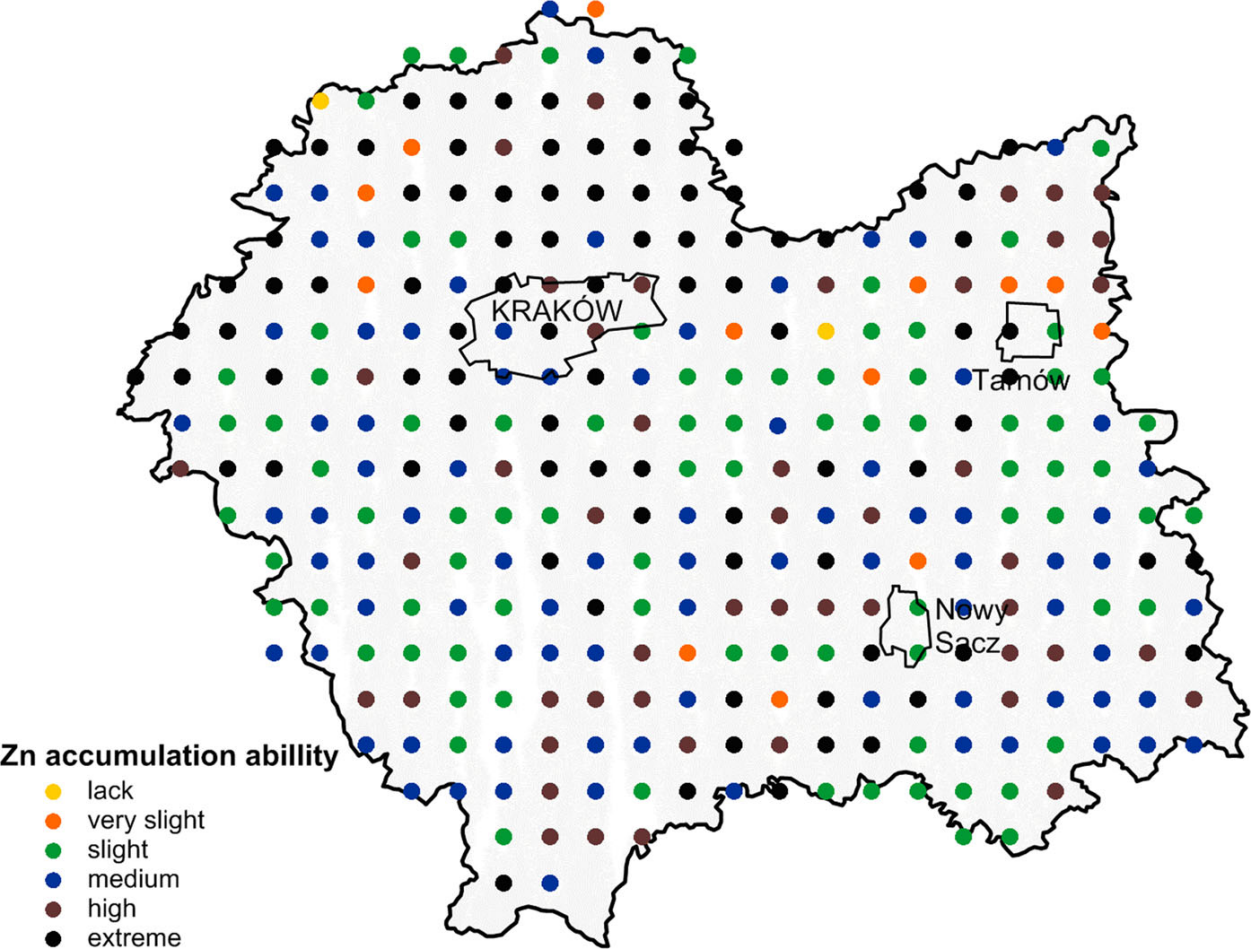
Distribution of sampling points and the ability to accumulation of zinc in soils

The binding capacity of the soil was taken into account when assessing the risk of Zn uptake by plants and other soil organisms and Zn entering the food chain. The soil–zinc binding ability, calculated for 320 sampling points, is shown in Fig. 4. These results show that 28% of soil samples had an extreme (class 5) zinc accumulation ability. Soil with a very high (class 4) zinc binding capacity constituted 15% of all the soil samples, whereas those with medium (class 3) and slight (class 2) capacity constituted 26% each. Only 5% of the soil samples had very slight (class 1) and lack (class 0) of zinc binding capacity (Fig. 4). Depending on the type of land use, the zinc binding capacity in arable land soils formed the following order: extreme (44%) > slight, medium (18% each) > high (15%) > very slight (5%); in the grassland: medium (30%) > extreme (26%) > slight, high (21% each) > very slight (2%); in the forest soil: slight (46%) > medium (23%) > very slight, extreme (10% each) > high (4%) > lack (2%); and in the wasteland: extreme (41%) > medium (30%) > slight (17%) > high (11%). The forest soils had the highest organic C content compared to arable land, grassland and wasteland. Moreover, soil samples from the forest area generally had a very acid and acid reaction and contained the highest amounts of sand. Spatial distribution of individual classes of the zinc binding capacity in the soils indicated that soils with a strong zinc binding capacity were predominant (61%) in the northwestern part of the Malopolska Province. However, in the southwestern parts of Malopolska, soils with slight and medium zinc accumulation ability were predominant. In the northeastern and southwestern part of the Malopolska Province, a high diversity of soil–zinc binding ability was observed (Fig. 4). Wieczorek and Baran (2013) found that the main reason for a slight zinc binding capacity in soils in the vicinity of the galvanizing plant was associated with prevalence of acid sandy soils in this area. Towers and Paterson (1997) indicated that soil pH is the main factor conditioning the metal-binding capacity of soil, but organic C content and clay content influence the final assessment.

### Assessment of the potential ecological risk of zinc in soils

The calculated values of the potential ecological risk and hazard quotient for zinc are summarized in Table 5. The *E*_*r*_^*i*^ for zinc ranged from 0.21 to 180.5 with a mean of 3.05 (Table 5). The results of *E*_*r*_^*i*^ calculations showed that 318 samples had a low potential ecological risk to the environment. The potential ecological risk was evaluated as moderate only for one sampling point, and only for one point was it evaluated as very strong. In terms of the mean potential ecological risk indices for four types of land use, the potential ecological risk is arranged in the following order: grassland > arable land > wasteland > forest soils (Table 5). The HQ values for zinc ranged from 0.03 to 24.07 (Table 5). In 10% of sampling points, HQ values indicated a potential harmful effect of zinc on ecological receptors. In 90% of sampling points, the HQ value was below one, which means lack of potential negative effects on ecological receptors. Depending on the type of land use, the HQ for Zn in the soils formed the following series: grassland > arable land > wasteland = forest soils (Table 5). The highest values of *E*_*r*_^*i*^ and HQ for Zn were observed in the northwestern part of the Malopolska Province, in the region near the mining–metallurgical activity involving processing of zinc and lead ores (Fig. 1). Our other studies revealed high values of the geoaccumulation index (*I*_geo_), pollution index (PI) and integrated pollution index (IPI) for heavy metals in soils in the northwestern part of the Malopolska Province (Baran et al. 2014; Baran and Wieczorek 2015). Zn and Pb ore mining and processing industry, neighborhood of metallurgical plants, municipal and industrial landfill sites and urban and communication areas are all sources of soil pollution in the northwestern part of the Malopolska Province. Apart from the above-mentioned anthropogenic factors, attention should be drawn to the fact that some soils in northwestern Malopolska have a naturally high content of heavy metals because these soils were formed from bedrocks containing considerable amounts of metals (Cabała and Teper 2007; Baran and Wieczorek 2015).

**Table 5:** Statistical results of potential ecological risk index and hazard quotient of Zn

Samples	Potential ecological risk index *E*_*r*_^*i*^			Hazard quotient (HQ)		
	Minimum	Mean	Maximum	Minimum	Mean	Maximum
Arable land	0.61	2.44	20.52	0.08	0.33	2.74
Grassland	0.21	4.33	180.5	0.03	0.58	24.07
Forest soils	0.45	2.11	11.07	0.06	0.28	1.48
Wasteland	0.50	2.12	10.48	0.07	0.28	1.40
All samples	0.21	3.05	180.5	0.03	0.41	24.07
Assessment	*E*_*r*_^*i*^ values and classification: *E*_*r*_ < 40—low; 40 ≤ *E*_*r*_ < 80—moderate; 80 ≤ *E*_*r*_ < 160—higher; 160 ≤ *E*_*r*_ < 320—much higher; 320 ≤ *E*_*r*_—serious^a^			HQ > 1 potential negative effects relative to the ecological receptors; HQ < 1—lack of potential negative effects to the ecological receptors^b^		

^a^Håkanson (1980) and Jiang et al. (2014)

^b^Swartjes et al. (2008) and Klimkowicz-Pawlas et al. (2012)

### PCA analyses

**Fig. 5:**
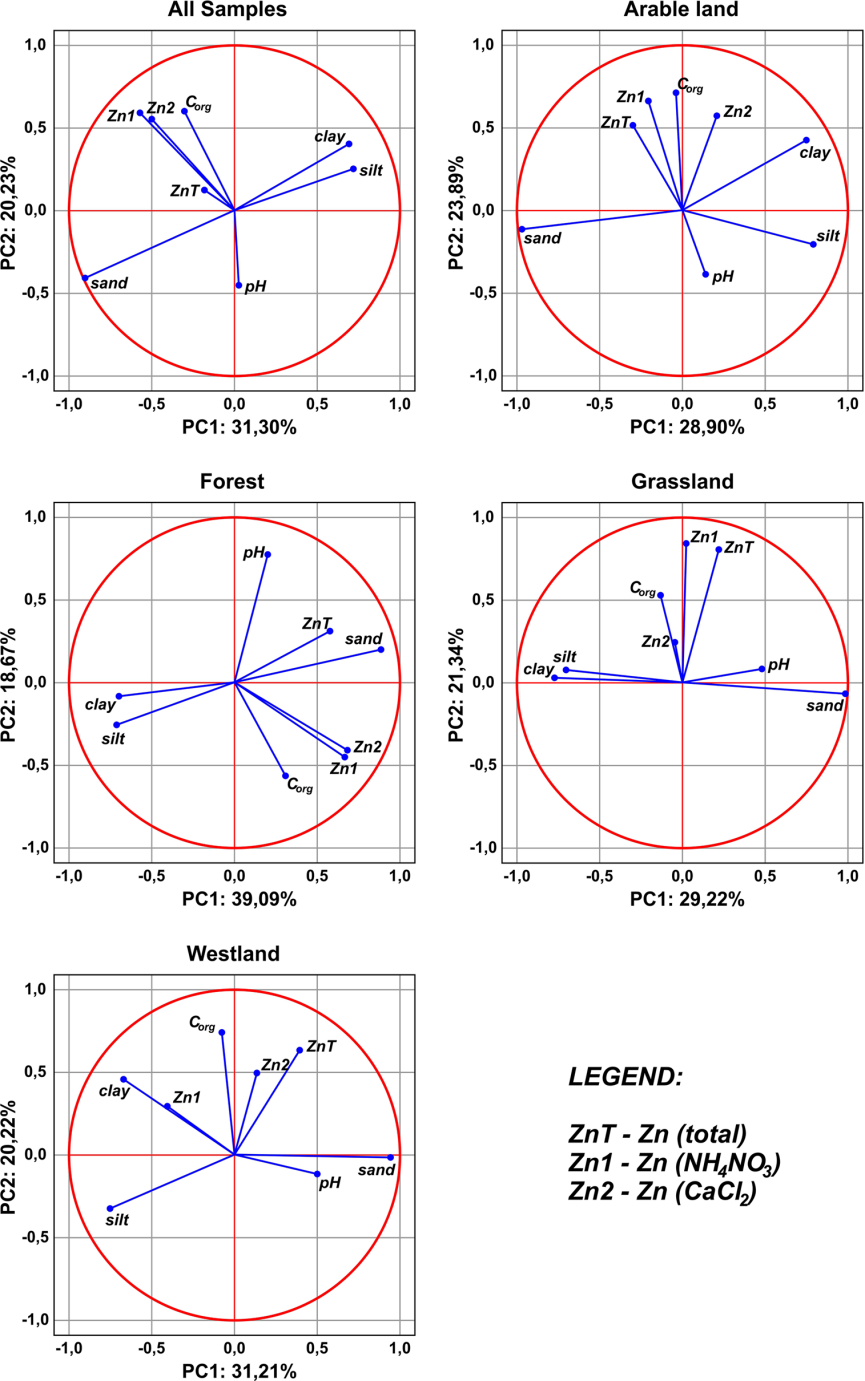
Results of PCA relationships between zinc and soil factors

Principal component analysis partially confirmed the results obtained from the analysis of correlation between the studied parameters (Fig. 5; Table 6). When analyzing all soil samples collected in Malopolska, very strong correlations between organic C content in these soils and bioavailable zinc content were found. It was also established that individual granulometric fractions are the main components of the first component, whereas organic C, bioavailable zinc and, partially, pH are components of the second component. What is more, it was shown that total zinc, which is the most important representative of the third principal component, is least represented by monoplot of the first two principal components. Principal component analysis was somewhat different in the case of application of classification which took into account the types of land use (Fig. 5). While the significance and correlations between the granulometric fractions did not change, an increasing role of total zinc in characterizing the overall variability in a set represented by first two principal components was observed, whereas the role of organic C was slightly reduced. Soil pH vector practically did not change on a monoplot of the first two principal variables for all types of land use except for the forest soils. The pH in the forest soils was the principal component of the second principal component. In the case of soils from grassland, the content of zinc (determined in CaCl_2_) was strongly displaced to further principal components. Moreover, a strongly positive correlation between total zinc and zinc determined in NH_4_NO_3_ was observed. In arable land, grassland and wasteland soils, no significant values of correlation between total zinc content and granulometric fractions were recorded. Regardless of the studied group of soils, the percent of overall variability, represented by the first two principal factors, was slightly more than 50%. The set of soil samples representing forest lands had the highest level of representation of the total data variance (PCA for the first two principal components was 57.76%), whereas the set of grassland soil samples had the lowest level of representation (PCA 50.56%) (Fig. 5).

**Table 6:** Component matrix for variables (*n* = 320)

Variables	PCA 1	PCA 2
C-organic	−0.302962	0.600437
Sand	−0.900261	−0.412847
Silt	0.717161	0.248886
Clay	0.692006	0.397191
Total zinc	−0.184748	0.122350
Zn (CaCl_2_)	−0.497301	0.546477
Zn (NH_4_NO_3_)	−0.571348	0.588521
pH	0.027404	−0.455902
% Of the total variance	31.30	20.23

## Conclusions

Generally, pollution of Malopolska soils with zinc is quite slight. Based on statistical and geostatistical analyses, the zinc content was likely affected by natural factors (soil factors) and the type of land use. In fact, a point source of contamination for Zn was noted in the study area. Soils polluted with Zn are located in the northwestern part of the province, near the mining–metallurgical activity involving processing of zinc and lead ores. These findings are confirmed by the arrangement of semivariogram surfaces and bivariate Moran’s correlation coefficients. There is no ecological risk of zinc to living organisms, only 10% of the soils indicated a potential harmful effect of zinc to ecological receptors. In 90% of the soils, there were no potentially negative effects of zinc to ecological receptors. Moreover, our results showed that 69% of the soil samples had extreme, very high and medium zinc accumulation ability. Slight and very slight zinc binding capacities were observed in 31% of the soil samples. According to our results, soil properties such as organic C, pH, sand, silt and clay content correlated with the content of bioavailable forms of zinc in the soils. However, the PCA suggests that organic C was the key factor to control bioavailability of zinc in the soils.

To sum up, the study of total zinc content as well as of bioavailable forms of this metal and soil–zinc binding ability is an important step in assessing the risk of potential transfer in the soil–plant–human chain and can improve the ecosystem and human health. The applied statistical and geostatistical method managed to reveal the pattern of zinc distribution in soils in various conditions. Our results can be used for planning, risk assessment and decision making in the environmental management in this region.

## References

[CR1] Alloway BJ (2009). Soil factors associated with zinc deficiency in crops and humans. Environmental Geochemistry and Health.

[CR2] Anju M, Banerjee DK (2011). Associations of cadmium, zinc, and lead in soils form lead and zinc mining area as studied by single and sequential extractions. Environmental Monitoring and Assessment.

[CR3] Anselin L (1995). Local indicators of spatial association–LISA. Geographical Analysis.

[CR4] Anselin L, Syabri I, Kho Y (2006). GeoDa: An introduction to spatial data analysis. Geographical Analysis.

[CR5] Baran A (2012). Assessment of zinc content and mobility in maize. Ecological Chemistry and Engineering A.

[CR6] Baran A (2013). Assessment of Zea mays sensitivity to toxic content of zinc in soli. Polish Journal of Environmental Studies.

[CR7] Baran A, Czech T, Wieczorek J (2014). Chemical properties and toxicity of soils contaminated by mining activity. Ecotoxicology.

[CR8] Baran A, Jasiewicz Cz (2013). Use of Phytotoxkit™ test in assessment of toxicity of soils polluted with zinc. Scientific Journal of Wroclaw University Environment and Life Sciences, Series of Agronomy.

[CR9] Baran A, Tarnawski M, Koniarz T (2016). Spatial distribution of trace elements and ecotoxicity of bottom sediments in Rybnik reservoir, Silesian-Poland. Environmental Science of Pollution Research.

[CR10] Baran A, Wieczorek J (2015). Application of geochemical and ecotoxicity indices for assessment of heavy metals content in soils. Archives Environment Protection.

[CR11] Blume HP, Brummer G (1991). Prediction of heavy metal behaviour in soil by means of simple field tests. Ecotoxicology and Environmental Safety.

[CR12] Bortey-Sam N, Nakayama SMM, Akoto O, Ikenaka Y, Baidoo E, Mizukawa H, Ishizuka M (2015). Ecological risk of heavy metals and metalloid in agricultural soils in Tarkwa, Ghana. International Journal of Environmental Research Public Health.

[CR13] Broadley MR, White PJ, Hammond JP, Zelko I, Lux A (2007). Zinc in plants. New Phytologist.

[CR14] Cabała J, Teper L (2007). Metalliferous constituents of rhizosphere soils contaminated by Zn–Pb mining in southern Poland. Water, Air, and Soil Pollution.

[CR15] Cabała J, Żogała B, Dubiel R (2008). Geochemical and geophysical study of historical Zn–Pb ore processing waste dump areas (Southern Poland). Polish Journal and Environmental Studies.

[CR16] Caetano AL, Marques CR, Goncalves F, de Silva EF, Pereira R (2016). Copper toxicity in a natural reference soil: Ecotoxicological data for the derivation of preliminary soil screening values. Ecotoxicology.

[CR17] Czech T, Gambuś F, Urbańska K, Wieczorek J (2014). Zinc uptake from soil at various times polluted with heavy metals. Ecology, Chemistry and Engineering A.

[CR18] Czech T, Gambuś F, Wieczorek J (2014). Mathematical forecasting methods for predicting lead contents in animal organs on the basis of the environmental conditions. Ecotoxicology and Environmental Safety.

[CR19] Degryse F, Broos K, Smolders E, Merckx R (2003). Soil solution concentration of Cd and Zn can be predicted with a CaCl_2_ soil extract. European Journal of Soil Science.

[CR20] Delavar MA, Safari Y (2016). Spatial distribution of heavy metals in soils and plants in Zinc Town, northwest Iran. International Journal of Environmental Science and Technology.

[CR21] Ettler V, Mihaljevic M, Sebek O, Grygar T (2007). Assessment of single extractions for the determination of mobile forms of metals in highly polluted soils and sediments: Analytical and thermodynamic approaches. Analytica Chimica Acta.

[CR22] Ewers W, Merian E (1991). Standards, guidelines and legislative regulatory concerning metals and their compounds. Metals and their compounds in the environment.

[CR23] Fang SB, Jia XB, Yang XY, Li YD, An SQ (2012). A method of identifying priority spatial patterns for management of potential ecological risks posed by heavy metals. Journal of Hazardous Materials.

[CR24] Fazeli MSh, Moosami MH, Pournia M, Zergani ZJ (2009). Metal distribution in topsoil around industrial town of Ahwaz II, Ahwaz, Iran. Journal of Applied Sciences.

[CR25] Fedotov PS, Koerdel W, Miró M, Peijnenburg WJGM, Wennrich R, Huang PM (2012). Extraction and fraction methods for exposure assessment of trace metals, metalloids and hazardous organic compounds in terrestrial environments. Critical Reviews Environmental Science and Technology.

[CR26] Guo G, Wu F, Xie F, Zhang R (2012). Spatial distribution and pollution assessment of heavy metals in urban soils from southwest China. Journal of Environmental Sciences.

[CR27] Håkanson L (1980). An ecological risk index for aquatic pollution control. A sedimentological approach. Water Research.

[CR28] Hani A, Pazira E (2011). Heavy metals assessment and identification of their sources in agricultural soils of Southern Teheran. Iran. Environmental Monitoring Assessment.

[CR29] Harmsen J (2007). Measuring bioavailability: Form a scientific approach to standard methods. Journal of Environmental Quality.

[CR30] Hill RAH, Chapman PM, Mann GS, Lawrance GS (2000). Level of detail in ecological risk assessment. Marine Environmental Bulletin.

[CR31] Jiang X, Lu WX, Zhao HQ, Yang QC, Yang ZP (2014). Potential ecological risk assessment and prediction of soil heavy metal pollution around gangue dump. Natural Hazards and Earth System Sciences.

[CR32] Journal of Laws of 2002, No. 165, item 1359. Regulation of the Minister of Environment of 9 September 2002 on soil and earth quality standards.

[CR33] Kabała C, Chodak T, Szerszeń L, Karczewska A, Szopka K, Frątczak U (2009). Factors influencing the concentration of heavy metals in soils of allotment gardens in the city of Wroclaw, Poland. Fresenius Environmental Bulletin.

[CR34] Kabata-Pendias A (2004). Soil–plant transfer of trace elements—an environmental issue. Geoderma.

[CR35] Kabata-Pendias A, Pendias H (2001). Trace elements in soils and plants.

[CR36] Khan MU, Muhammad S, Malik RN (2013). Potential risk assessment of metal consumption in food crops irrigated with wastewater. CLEAN – Soil, Air, Water.

[CR37] Kim RY, Yoon JK, Kim TS, Yang JE, Owens G, Kim KR (2015). Bioavailability of heavy metals in soils: Definitions and practical implementation: A critical review. Environmental Geochemistry and Health.

[CR38] Kizilkaya R, Dengiz O, Ozyazici MA, Askin T, Mikayilov F, Shein EV (2011). Spatial distribution of heavy metal in soils of the Bafra Palin in Turky. Eurasian Soil Science.

[CR39] Klimkowicz-Pawlas A, Maliszewska-Kordybach B, Smreczak B (2012). Application of preliminary stage of risk assessment procedure for agricultural soils. Area affected by flood as a case study. Journal and Food Agriculture Environment.

[CR40] Krami KL, Amri F, Sefiyanian A, Shariff ARBM, Tabatabaie T, Pradhan B (2013). Spatial patterns of heavy in soil under different geological structures and land uses for assessing metal enrichments. Environment Monitoring Assessment.

[CR41] Lago-Vila M, Rodriguez-Sejio A, Arenas-Lago D, Andrade L, Vega MFAV (2016). Heavy metal content and toxicity of mine and quarry soils. Journal Soils and Sediments.

[CR42] Liu M, Li Y, Zhang W, Wang Y (2013). Assessment and spatial distribution of zinc pollution in agricultural soils of Chaoyang, China. Procedia Environmental Sciences.

[CR43] Liu G, Xue W, Tao L, Liu X, Hou J, Wilton M, Gao D, Wang A, Li R (2013). Vertical distribution and mobility of heavy metals in agricultural soils along Jishui River affected by mining in Jiangxi province, China. CLEAN – Soil, Air, Water.

[CR44] Meers E, Samson R, Tack FMG, Ruttens A, Vandegehuchte M, Vangronsveld J, Verloo MG (2007). Phytoavailability assessment of heavy metals in soils by single extractions and accumulation by *Phaseolus vulgaris*. Environmental and Experimental Botany.

[CR45] Mucha J, Wasilewska-Błaszczyk M (2015). Geostatistical support for categorization of metal ore resources in Poland. Mineral Resources Management.

[CR46] Olaniran AO, Balgobind A, Pillay B (2013). Bioavailability of heavy metals in soil: Impact on microbial biodegradation of organic compounds and possible improvement strategies. International Journal of Molecural Sciences.

[CR47] Pueyo M, Lopez-Sanchez JF, Rauret G (2004). Assessment of CaCl_2_, NaNO_3_, and NH_4_NO_3_ extraction procedures for the study of Cd, Cu, Pb and Zn extractability in contaminated soils. Analytica Chimica Acta.

[CR48] Rauret G (1998). Extraction procedures for the determination of heavy metals in contaminated soil and sediment. Talanta.

[CR49] Reichman SM (2002). The responses of plants to metal toxicity: A review focusing on copper, manganese and zinc. Australia Mineral and Energy Environment Foundation.

[CR50] Romero-Freire A, García Fernandez I, Simon Torres M, Martínez Garzon FJ, Martín Peinado FJ (2016). Long-term toxicity assessment of soils in a recovered area affected by a mining spill. Environ Pollution.

[CR51] Rutkowska B, Szulc W, Bomze K, Gozdowski D, Spychaj-Fabisiak E (2015). Soil factors affecting solubility and mobility of zinc in contaminated soils. International Journal of Environmental Sciences and Technology.

[CR52] Sagardoy R, Morales F, López-Millán AF, Abadía A, Abadía J (2009). Effects of zinc toxicity on sugar beet (*Beta vulgaris* L.) plants grown in hydroponics. Plant Biology.

[CR53] Saito H, Goovaerts P (2000). Geostatistical interpolation of positively skewed and censored data in a dioxin-contaminated site. Environmental Sciences and Technology.

[CR54] Shaheen SM, Rinklebe J (2014). Geochemical fractions of chromium, copper, and zinc and their vertical distribution in floodplain soil profiles along the Central Elbe. Geoderma.

[CR55] Solomon KR, Sibley P (2002). New concepts in ecological risk assessment: Where do we go from here?. Marine Pollution Bulletin.

[CR56] Sterckeman T, Douay F, Proix N, Fourrier H (2000). Vertical distribution of Cd, Pb and Zn in soils near smelters in the North of France. Environmental Pollution.

[CR57] Sun Y, Zhou Q, Xie X, Liu R (2010). Spatial, sources and risk assessment of heavy metal contamination of urban soils in typical regions of Shenyang, China. Journal of Hazardous Materials.

[CR58] Swartjes FA, Carlon C, De Wit NHSM (2008). The possibilities for the EU—wide use of similar ecological risk—based soil contamination assessment tools. Science of the Total Environment.

[CR59] Towers W, Paterson E (1997). Sewage sludge application to land—a preliminary assessment of the sensitivity of Scottish soils to heavy metal inputs. Soils Use Management.

[CR60] Ure A, Quevauviller P, Muntau H, Griepink B (1993). Speciation of heavy metals in soils and sediments. An account of the improvement and harmonization of extraction techniques undertaken under auspices of the BCR of the Commission of the European Communities. International Journal of Environmental Analytical Chemistry.

[CR61] Wieczorek J, Baran A (2013). Assessment of possible zinc accumulation in soils in the zone of possible zinc accumulation in soils in the zone of potential zinc-works influence. Ecological Chemistry and Engineering A.

